# Synthesis-dependent repair of Cpf1-induced double strand DNA breaks enables targeted gene replacement in rice

**DOI:** 10.1093/jxb/ery245

**Published:** 2018-06-28

**Authors:** Shaoya Li, Jingying Li, Jiahui Zhang, Wenming Du, Jindong Fu, Suhas Sutar, Yunde Zhao, Lanqin Xia

**Affiliations:** 1 Institute of Crop Sciences (ICS), Chinese Academy of Agricultural Sciences (CAAS), Beijing, China; 2 Section of Cell and Developmental Biology, University of California, San Diego, La Jolla, CA, USA; 3 National Key Laboratory of Crop Genetic Improvement and National Center of Plant Gene Research (Wuhan), Huazhong Agricultural University, Wuhan, China

**Keywords:** Cpf1, gene replacement, homology-directed DNA repair (HDR), ribozyme, rice (*Oryza sativa* L), synthesis-dependent strand annealing (SDSA)

## Abstract

The recently developed CRISPR (clustered regularly interspaced short palindromic repeats)/Cpf1 system expands the range of genome editing and is emerging as an alternative powerful tool for both plant functional genomics and crop improvement. Cpf1-CRISPR RNA (crRNA) produces double strand DNA breaks (DSBs) with long 5'-protruding ends, which may facilitate the pairing and insertion of repair templates through homology-directed repair (HDR) for targeted gene replacement and introduction of the desired DNA elements at specific gene loci for crop improvement. However, the potential mechanism underlying HDR of DSBs generated by Cpf1-crRNA remains to be investigated, and the inherent low efficiency of HDR and poor availability of exogenous donor DNA as repair templates strongly impede the use of HDR for precise genome editing in crop plants. Here, we provide evidence of synthesis-dependent repair of Cpf1-induced DSBs, which enables us precisely to replace the wild-type *ALS* gene with the intended mutant version that carries two discrete point mutations conferring herbicide resistance to rice plants. Our observation that the donor repair template (DRT) with only the left homologous arm is sufficient for precise targeted allele replacement offers a better understanding of the mechanism underlying HDR in plants, and greatly simplifies the design of DRTs for precision genome editing in crop improvement.

## Teaser

Synthesis-dependent strand annealing is a primary mechanism in the repair of Cpf1-induced DSBs on homology repair templates; such templates are available and enable targeted gene replacement in rice.

## Introduction

Double strand DNA breaks (DSBs) in target genes generated by Cas endonucleases are repaired by the error-prone non-homologous end joining (NHEJ) pathway or the precise homology-directed repair (HDR), or both NHEJ and HDR (Jinek *et al.*, 2012; Cong *et al.*, 2013; Zetsche *et al.*, 2015). NHEJ often leads to a small insertion/deletion and is widely used in generating loss-of-function mutants. HDR utilizes a DNA donor repair template (DRT) that is flanked with sequences homologous to those adjacent to the DSBs to guide the DNA repair (Puchta, 1998). HDR can be used for targeted allele replacement or introduction of markers to target loci, providing a valuable tool for crop improvement. In principle, three potential mechanisms have been proposed for HDR of DSBs: single-strand annealing (SSA), synthesis-dependent strand annealing (SDSA), and the so-called double strand break repair (DSBR) model (Puchta, 1998). During SSA, both ends of the DSB carry complementary sequences. These molecules can then anneal to one another to form a chimeric DNA molecule with the 3' overhangs. Consequently, the sequences between the complementary sequences will be lost. Therefore, SSA is also classified as a non-conservative HDR-mediated DSB repair mechanism (Puchta and Fauser, 2013). In the cases of DSBR and SDSA, the homologous repair template can be supplied in *cis* or in *trans*. Following the DSB induction, 3' end invasion of a single strand into a homologous double strand occurs, resulting in a D-loop. Reparative synthesis is initiated using the newly paired strand as a template. In DSBR, DNA synthesis occurs at both broken ends so that genetic information is copied from both strands of the homologous sequences, which may lead to a crossover event (Puchta and Fauser, 2013). DSBR is a prominent mechanism for meiotic recombination (Osman *et al.*, 2011). In SDSA, the DSB is at first resected and processed to generate 3' overhangs on both sides of the DSB. The 3' overhangs are then paired with the homologous arms of the DRT and are extended by DNA synthesis using the DRT as a template. Then, the newly synthesized strands withdraw from the DRT and anneal back to the locus (Puchta and Fauser, 2014; Paix *et al.*, 2017). SDSA has been proposed as a repair mechanism when the single-stranded oligo DNA nucleotides (ssODNs) are used as the DRT (Paquet *et al.*, 2016; Richardson *et al.*, 2016; Kan *et al.*, 2017). When dsDNAs are used as repair templates, different DSBs generated by Cas9 variants engage in different repair pathways and the polarity of the overhang structure is a critical determinant of DSB repair pathway choice in human cells (Bothmer *et al.*, 2017). However, analyses of the sequence requirements for efficient repair of DSBs generated by Cas9 in human cells indicated that the repair process is more consistent with SDSA when either ssODNs or dsDNAs are used as repair templates (Paix *et al.*, 2017). Thus, the mechanism underlying HDR of DSBs when a dsDNA is used as the DRT still remains inconclusive, especially in plants.

Genome editing technologies enable precise modifications of DNA sequences *in vivo* and offer great promise for crop improvement. CRISPR/Cas9 (clustered regularly interspaced short palindromic repeats/CRISPR-associated Cas9) has revolutionized genome editing because of its simplicity and versatility (Cong *et al.*, 2013; Miao *et al.*, 2013; Shan *et al.*, 2013; Ma *et al.*, 2015; Sun *et al.*, 2016; J. Li *et al.*, 2018). CRISPR/Cpf1 (CRISPR from *Prevoltella* and *Francisella* 1), a new class 2 CRISPR/Cas system, was recently exploited as an alternative to the widely used SpCas9 in genome editing in many organisms including plants (Zetsche *et al.*, 2015, 2017; Fonfara *et al.*, 2016; Kim *et al.*, 2016; Gao *et al.*, 2017; Hu *et al.*, 2017; Kim *et al.*, 2017; Tang *et al.*, 2017; Wang *et al.*, 2017; Xu *et al.*, 2017; S. Li *et al.*, 2018; Zhong *et al.*, 2018). Cpf1 utilizes a thymidine-rich protospacer adjacent motif (PAM) site ‘TTTN’ (Zetsche *et al.*, 2015) or non-canonical ‘TYCV’ (S. Li *et al.*, 2018), and is guided by a single CRISPR RNA (crRNA) (Zetsche *et al.*, 2015). The Cpf1 crRNA (~43 nt) is shorter than that of SpCas9 single guide RNA (sgRNA) by 60 nucleotides and no *trans*-acting crRNA is needed (Fonfara *et al.*, 2016). Moreover, Cpf1 produces long 5'-protruding ends, which may facilitate the pairing and insertion of repair templates (Zetsche *et al.*, 2015). Thus, the CRISPR/Cpf1 system is emerging as an attractive tool for editing AT-rich regions (Begemann *et al.*, 2017; Tang *et al.*, 2017; Wang *et al.*, 2017; S. Li *et al.*, 2018). Herein, we coupled a plant codon-optimized LbCpf1 from *Lachnospiraceae bacterium* ND 2006 (Wang *et al.*, 2017) with a simple ribozyme crRNA array driven by the *OsU3* promoter (Gao and Zhao, 2014). We demonstrated that DRTs with either only the left homologous arm or two homologous arms function efficiently in achieving precise targeted replacement of the *Acetolactate synthase* (*ALS*) gene, which encodes a key enzyme for the biosynthesis of the branched chain amino acids leucine, isoleucine, and valine, and is a major target for ALS-inhibiting herbicides such as chlorsulfuron and bispyribac sodium (BS) in rice (Mazur *et al.*, 1987). Our findings support the hypothesis that SDSA is involved in HDR of DSBs generated by Cpf1-crRNA in plant cells when the dsDNA is used as a DRT. Our observation that the left homologous arm alone is sufficient for HDR not only offers a better understanding of the mechanism underlying HDR in plants, but also greatly simplifies the design of DRTs for targeted allele replacement in crop plants.

## Materials and methods

### Construction of the CRISPR/LbCpf1-related vectors

The vectors used in this study were based on the vector pCXUN-LbCpf1 that replaced the ubiquitin-Cas9 in plasmid pCXUN-Cas9 (Sun *et al.*, 2016) with ubiquitin-LbCpf1 in plasmid LbCpf1-OsU6 (Wang *et al.*, 2017). The backbone of pCXUN-LbCpf1 contains a hygromycin-resistant gene (*hpt*) for callus selection. The *Sac*I and *Pme*I sites in pCXUN-LbCpf1 were used for introducing the OsU3–RCR1–RCR2 expression cassette and the DRT, respectively (Supplementary Fig. S1 at *JXB* online).

The RCR1 unit was assembled through two rounds of overlapping PCRs. The first PCR was performed using primer set RCR1F2/RCR-common-R with the plasmid pRS316-RCR-GFP (Zhang *et al.*, 2017) as a template, and the second one used primer set RCRF1/RCR-common-R using the PCR1 product as a template (Supplementary Table S1). The same procedure was used to obtain the RCR2 unit by utilizing the primers RCR2-F2/RCR-common-R and RCR-F1/RCR-common-R, respectively (Supplementary Table S1). The *OsU3* promoter sequence was amplified with primer set OsU3F/OsU3R using plasmid pCXUN-Cas9-OsU3 as template (Supplementary Table S1). Because *OsU3* promoter sequences were used in this experiment, we also placed an A before the first nucleotide of the target sequences. The full-length OsU3–RCR1–RCR2 cassette was obtained through five rounds of overlapping PCRs. The first PCR was performed with primer set OsU3F/OsU3-RCR1R using the *OsU3* promoter sequence as template (Supplementary Table S1). The second PCR was performed with primer set RCR-Common-F/RCR1-10 random-R using the RCR1 unit as the template (Supplementary Table S1). Products of PCRs 1 and 2 were used as templates for the third PCR with the primer set OsU3-F/RCR1-10 random-R to generate the OsU3–RCR1 cassette (Supplementary Table S1). The fourth PCR was performed with primer set RCR2-10 random-F/SacI-RCR2-R using the RCR2 unit as the template (Supplementary Table S1). The products of PCRs 3 and 4 were used as templates for the fifth PCR with the primer set SacI-OsU3-F/SacI-RCR2-R to generate the OsU3–RCR1–RCR2 cassette. At the 5' end of the primer pair SacI-OsU3-F/SacI-RCR2-R, the sequences are homologous to the sequences outsides of the *Sac*I site in pCXUN-LbCpf1. Then, the OsU3–RCR1–RCR2 fragment was cloned to the linearized pCXUN-LbCpf1 with *Sac*I, by using the pEASY-Uni Seamless Cloning and Assembly Kit (TransGen Biotech, Beijing, China). The vector harboring OsU3–RCR1–RCR2 was named pCXUN-LbCpf1-OsU3-RCR1-RCR2.

**Fig. 1.**
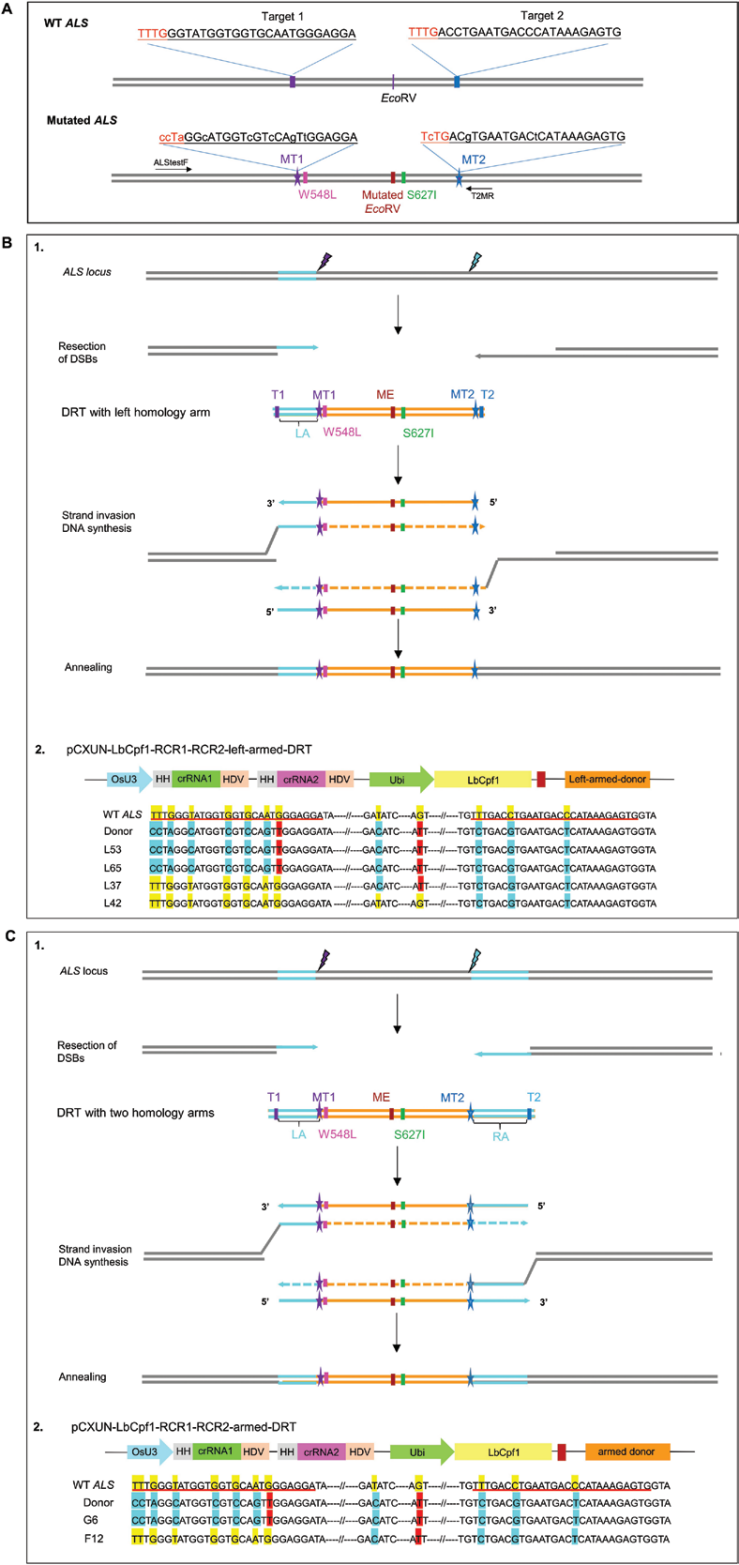
Homology-directed repair (HDR) of double strand breaks (DSBs) generated by the LbCpf1 endonuclease in rice. (A) A schematic description of HDR-mediated precise gene replacement in rice. A wild-type (WT) *ALS* gene fragment is removed by LbCpf1 and a couple of crRNAs. The LbCpf1 target sequences are underlined, and the PAM sites and the mutated PAM sites are underlined and highlighted in red. The WT *ALS* fragment is replaced by a mutant version that introduces changes of two discrete amino acid residues. The PAM sites and an *Eco*RV site are modified to prevent the replacement from further cleavage by LbCpf1/crRNAs and to facilitate detection of gene replacement events, respectively. (B) CRISPR/Cpf1-mediated HDR in rice calli using vector pCXUN-LbCpf1-OsU3-RCR1-RCR2-left-armed-DRT co-introduced with free left armed-DRT through CRISPR/Cpf1-mediated HDR. (B-1) A schematic description of the synthesis-dependent strand annealing (SDSA) pathway of HDR with the donor containing only the left homology arm. Each line corresponds to a DNA strand. Chromosomal DNA is in gray, DRT in orange, homology arms of DRT are in blue, and arrows refer to the 3' ends. T1, target 1; T2, target 2; MT1, mutated PAM site and mutated target 1; MT2, mutated PAM site and mutated target 2; LA, left arm; ME, mutated *EcoR*V. Resecting DSB creates 3' overhangs on each side of the DSB. The overhangs in the 3' end pair with complementary strands in the DRT and are extended. The newly synthesized strands withdraw from the donor and anneal back at the locus. (B-2) Sequence analyses of the representative HDR events. PCR products were amplified by allele-specific primer set ALStestF/T2MR as described in (A). The calli L53 and L65 had undergone precise HDR, whereas the calli L37 and L42 had undergone partial HDR. The sequences shadowed in yellow and blue represent the same bases as those of the wild type and the designed DRT, respectively. Specifically, the sequences shadowed in red indicate the expected targeted substitution. (C) CRISPR/Cpf1-mediated HDR in rice calli using vector pCXUN-LbCpf1-OsU3-RCR1-RCR2-armed-DRT co-introduced with free armed-DRT through CRISPR/Cpf1-mediated HDR. (C-1) A schematic description of the synthesis-dependent strand annealing (SDSA) pathway of HDR with a DRT containing two homologous arms. In this, and all other schematics, each line corresponds to a DNA strand. Chromosomal DNA is indicated in gray, DRT is in orange, the homology arms are in blue, and arrows indicate the 3' ends. T1, target 1; T2, target 2; MT1, mutated PAM site and mutated target 1; MT2, mutated PAM site and mutated target 2; LA, left arm; RA, right arm; ME, mutated *Eco*RV. (C-2) Sequence analyses of the representative HDR events. PCR products were amplified by allele-specific primer set ALStestF/T2MR as described in (A). The callus G6 had a precise HDR, whereas the callus F5 had undergone partial HDR. The sequences shaded in yellow and blue represent the wild-type and the designed donor repair template, respectively. Specifically, the sequences shaded in red indicate the expected targeted substitutions.

The DRT with a mutated *ALS* sequence was synthesized by BGI (Beijing Genomics Institute-Shenzhen, Chinese Academy of Sciences, Shenzhen, China) (named as armed-DRT) (Supplementary Fig. S2B). The vector pCXUN-LbCpf1-OsU3-RCR1-RCR2-left-armed-DRT was obtained by overlapping PCRs. The first PCR was performed with primer set donor-armLF/donor-armLR1 using the synthesized armed-donor sequence as template. The second PCR was performed with primer set donor-armLF/donor-armLR2 using the product of PCR1 as the template. Products of PCR 2 were used as templates for the third PCR with the primer set pme-donor-armLF/pme-donor-armLR to generate the donor with only the left homology arm (Supplementary Fig. S2A). This fragment was cloned into the *Pme*I site of pCXUN-LbCpf1-OsU3-RCR1-RCR2 by using the Assembly Kit (TransGen Biotech). The final plasmid was named pCXUN-LbCpf1-OsU3-RCR1-RCR2-left-armed-DRT (Fig. 1A). PCR primers for vector construction are listed in Supplementary Table S1.

For the vector pCXUN-LbCpf1-OsU3-RCR1-RCR2-armed-DRT, the donor fragment (Supplementary Fig. S2B) was amplified by PCR using primer set Pme-donorF/Pme-donorR with synthesized armed-DRT as template and cloned into the *Pme*I site of pCXUN-LbCpf1-OsU3-RCR1-RCR2 by using the Assembly Kit (TransGen Biotech). The final plasmid was named pCXUN-LbCpf1-OsU3-RCR1-RCR2-armed-DRT (Fig. 1B). PCR primers for vector construction are listed in Supplementary Table S1.

### *In vivo* CRISPR/Cpf1-mediated HDR in rice calli

In order to provide enough donor fragments for HDR, both vectors and the free 549 bp left homology armed-DRT and the 670 bp armed-DRT with two homologous arms were co-introduced into rice (*japonica* cv. Zhonghua 11) calli with a molar ratio of 1:20 by particle bombardment Two days after bombardment, DNA from the calli was extracted using a DNA Quick Plant System (Tiangen, Beijing, China). PCR amplification was performed using EASY Taq polymerase (TransGen Biotech) employing 200 ng of genomic DNA as template. Each callus was tested individually by PCR and sequencing. The PCR products were generated using the allele-specific primer set ALSTestF/T2MR (Supplementary Table S1) with the upstream primer located in the genome sequence of the *ALS* gene outside of the left homology arm, whereas the downstream primer was an allele-specific primer (Fig. 1A). The obtained amplicons were cloned into the cloning vector pEasy-Blunt Zero (TransGen Biotech). At least 10 positive colonies for each sample were sequenced.

### Rice transformation

The plasmids pCXUN-LbCpf1-OsU3-RCR1-RCR2-left-armed-DRT and pCXUN-LbCpf1-OsU3-RCR1-RCR2-armed-DRT were linearized by *Sac*II and then mixed with the free DRTs at a molar ratio of 1:20, which were co-transformed into calli of a *japonica* rice (cv. Zhonghua 11) by particle bombardment following the protocol described previously (Li *et al.*, 1993). Particle bombardments were performed using a PDS1000/He particle bombardment system (Bio-Rad, Hercules, CA, USA).

Rice calli induced from mature seeds were used for bombardment. After bombardment, the calli were selected on medium containing 50 mg l^–1^ hygromycin for 2 weeks to allow the growth of calli with the construct, either transiently expressed or stably integrated. Then the well-grown calli were transferred to induction medium with 0.4 μM BS. After 2 weeks, the vigorously grown calli were transferred to regeneration media to generate green plants.

### Molecular characterization of the edited plants

Rice genomic DNA from ~0.2 g of leaf tissue was extracted using a DNA Quick Plant System (Tiangen). PCR amplification was performed using EASY Taq polymerase (TransGen Biotech) and 100 ng of genomic DNA as a template. All plants were tested individually with PCR-RE (PCR and restriction enzyme digestion assay) and sequencing. The PCR products amplified by the primer pair ALSTestF/ALSTestR (Supplementary Table S1) were digested with *Eco*RV and then directly sequenced to screen for the plants with a modified *ALS* gene. The sequence chromatograms were analyzed by a web-based tool (http://skl.scau.edu.cn/dsdecode/) to confirm the genotype and zygosity of the tested plants (Liu *et al.*, 2015). Some PCR products were also cloned into the cloning vector P-easy (TransGen Biotech), and at least 10 positive colonies for each sample were sequenced. Primers for detection of the presence of *LbCpf1*, *crRNA*, and *hptII* are listed in Supplementary Table S1.

To investigate off-target effects, we selected three and two potential off-target sites based on the predictions of the Cas9-OFFinder tool (http://rgenome.net/cas-offinder/), for the target 1 and target 2, respectively (Supplementary Table S3). Site-specific genomic PCR and Sanger sequencing was used to determine the off-target effects. The primer sets are as listed in Supplementary Table S1.

## Results

### Left armed-DRT successfully mediated HDR in rice calli

**Fig. 2.**
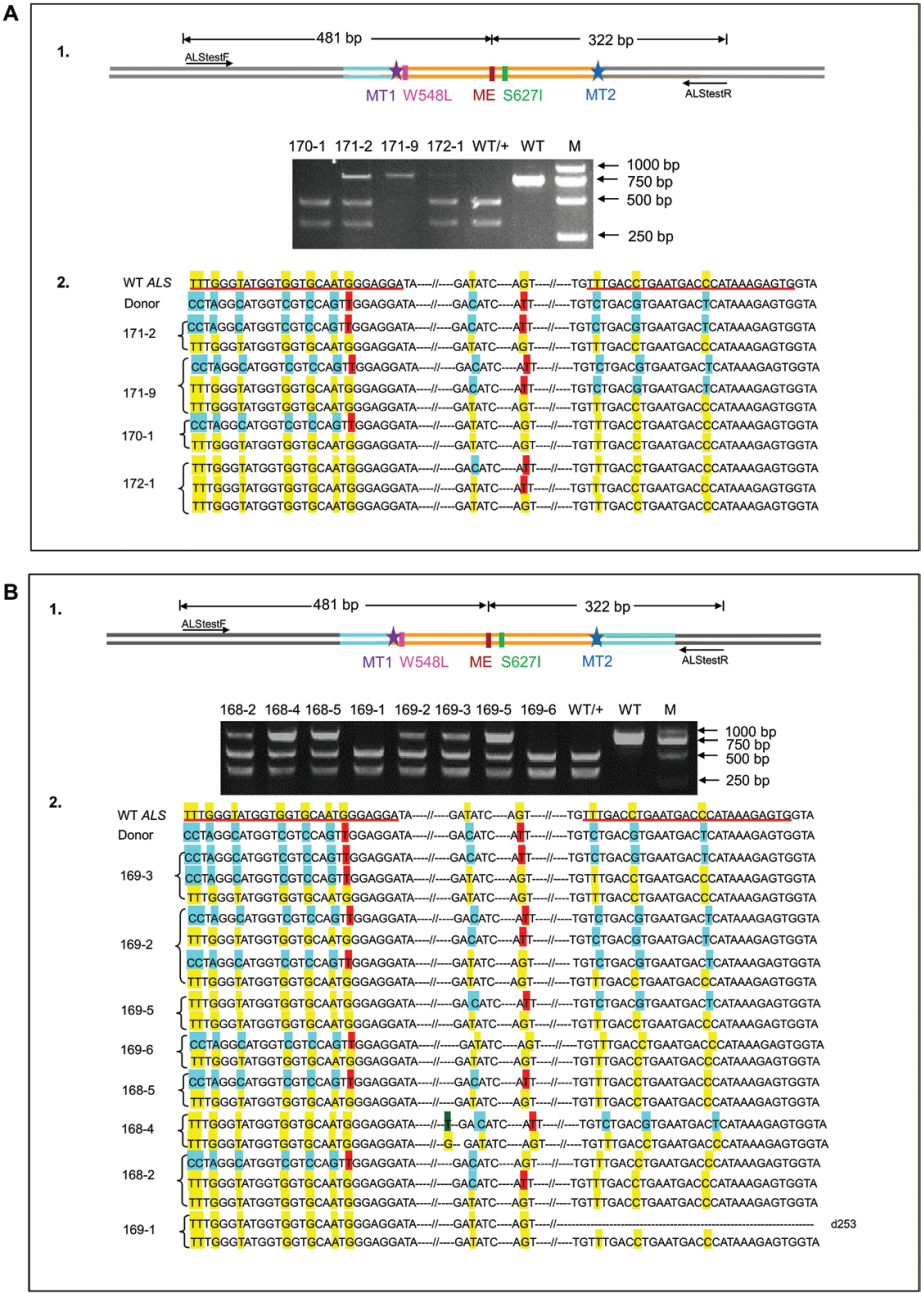
Generation of stable precisely edited rice lines through SDSA-mediated HDR. (A) Only the left homology arm strategy for CRISPR/LbCpf1-mediated HDR in rice. (A-1) PCR-RE analyses of the different genotypes. PCR products amplified by primers ALStestF/R were digested with *Eco*RV (GACATC). M, DL2000; WT, wild-type; T1, target 1; T2, target 2; MT1, mutated PAM site and mutated target 1; MT2, mutated PAM site and mutated target 2; ME, mutated *Eco*RV; WT/+, *Eco*RV cuts the PCR products of the wild type, resulting in 481 bp and 322 bp fragments. Successful HDR leads to *Eco*RV-resistant bands. (A-2) Sequence analyses of the HDR events. Line 171-2 has one allele with precise HDR, while the other is wild type. Line 171-9 is a chimeric line with three alleles: a precise HDR, a partial HDR, and a wild type. Line 170-1 has a heterozygous partial HDR. Line 172-1 is a chimeric line with three alleles: one base pair substitution, a partial HDR, and a wild type. The sequences shaded in yellow and blue represent wild-type and the designed donor repair template, respectively. Specifically, the sequences shaded in red indicate the expected targeted substitution. (B) Generation of stable precisely edited rice lines using pCXUN-LbCpf1-OsU3-RCR1-RCR2-armed donor vector through CRISPR/Cpf1-mediated HDR. (B-1) PCR-RE analyses of the different genotypes. PCR products amplified by primers ALStestF/R were digested with *Eco*RV (GACATC). M, DL2000; WT, wild-type; T1, target 1; T2, target 2; MT1, mutated PAM site and mutated target 1; MT2, mutated PAM site and mutated target 2; ME, mutated *Eco*RV; WT/+, *Eco*RV cuts the PCR products of the wild type, resulting in 481 bp and 322 bp fragments. *Eco*RV failed to digest the PCR products if HDR was successful. (B-2) Representative sequences of the different genotypes. Line 169-3 is a chimeric line with three alleles: one precise HDR, one partial HDR, and a wild type. Line 169-2 is a chimeric line with four alleles: a precise HDR, two partial HDR events, and a wild type. Lines 168-4, 168-5, 169-5, and 169-6 have one partial HDR, except for a synonymous G to T base substitution at position 1911 bp in Line 168-4. The 168-2 is a chimeric line with three alleles. The first and second allele have partial HDR, while the third allele is wild type. Line 169-1 has a 253 bp deletion. The sequences shaded in yellow and blue represent wild-type and the designed donor repair template, respectively. Specifically, the sequences shaded in red indicate the expected targeted substitutions. In d#, the # refers to the number of base pairs deleted from the target sites. Different numbers of lines indicate independent lines developed from resistant calli.

To investigate whether dsDNA with only a left homologous arm can effectively mediate HDR, we first tested this strategy in a transient assay in rice calli to replace the wild-type *ALS* gene with the mutated version (Fig. 1A). We designed a plasmid named pCXUN-LbCpf1-OsU3-RCR1-RCR2-left-armed-DRT, which harbors cassettes expressing LbCpf1, two crRNAs, and a designed DRT with only a 97 bp left homologous arm (left armed-DRT) (Fig. 1B-1; Supplementary Figs S1A, 2A). The two crRNAs could cut out a fragment from the wild-type *ALS* gene in the presence of LbCpf1. The 549 bp DRT (Supplementary Fig. S2A) contained two desired mutations (W548L and S627I) that rendered rice plants resistant to multiple ALS-inhibiting herbicides (Mazur *et al.*, 1987). We also introduced synonymous mutations into the two crRNA target sequences and a restriction site inside the DRT. We then flanked the DRT with the same two crRNA target sequences to enable the release of DRT from the vector *in vivo* (Supplementary Fig. S2A). Both the vector and the free 549 bp left armed-DRT were co-introduced into rice (*japonica* cv. Zhonghua 11) calli with a molar ratio of 1:20 by particle bombardment in order to enrich the availability of DRTs. After 2 d, genomic DNA was extracted from the bombarded calli to determine whether the intended gene replacement through SDSA was achieved following SDSA-mediated HDR with only left armed-DRT (Fig. 1B-1). By using the allele-specific primer set ALSTestF/T2MR (Table S1), we detected the precise replacement of the wild-type *ALS* gene with the mutant version (Fig. 1B-2). We also observed partial HDR events, by which only one end was replaced (Fig. 1B-2), suggesting that there might be template switching during the repair of DSBs (Smith *et al.*, 2007).

### HDR in rice calli mediated by DRT with two homologous arms

Similarly, we designed a plasmid named pCXUN-LbCpf1-OsU3-RCR1-RCR2-armed-DRT that harbors cassettes expressing LbCpf1, two crRNAs, and a designed DRT with two homologous arms (armed-DRT) (Figs 1C-1; Supplementary Fig. S1B). This 670 bp armed-DRT was the same as the 549 bp left armed-DRT shown above, except that it also has a 121 bp right homologous arm (Supplementary Fig. S2B). Following SDSA (Fig. 1C-1), we observed the intended precise replacement of the wild-type *ALS* gene with the mutant version and partial homologous recombination events when this armed-donor was used (Fig. 1C-2).

### Generation of stable edited rice plants by HDR using either left armed-DRT or DRT with two arms

We then investigate whether we can generate stable precisely edited rice lines using the two strategies described above. For vectors with left armed-DRT and those with armed-DRT, 152 and 164 calli were bombarded, respectively. The calli that survived one round of selection pressure of hygromycin were subsequently transferred onto induction medium and regeneration medium with 0.4 µM BS. Then, the regenerated plants were used for PCR-RE. PCR primer set ALStestF/R, which is located at the genome site beyond the two homologous arms in DRT, was designed to amplify an *ALS* fragment from both the wild-type *ALS* locus and the edited *ALS*, but not from the plasmid (Fig. 2; Supplementary Table S1). All plantlets developed from one callus were treated as a pool. The PCR products were then digested with *Eco*RV. The plantlets in a pool that gave a PCR-RE result different from that of the wild type were then transferred into soil individually and were tested with PCR-RE and sequenced. No obvious phenotypic variations were observed between these lines and the non-transformed wild-type control. In total, 71 and 94 plants developed from 15 and 20 resistant calli for the two different vectors, respectively, were selected for further analyses (Supplementary Table S2).

For vector pCXUN-LbCpf1-OsU3-RCR1-RCR2-left-armed-DRT, among the 71 plants recovered, PCR-RE and sequencing analyses identified one heterozygous line (Line 171-2) and one chimeric line (Line 171-9) with one allele containing the precise replacement as designed, and two lines (Lines 170-1 and 172-1) with partial HDR at either the W548L or the S627I locus (Fig. 2A; Supplementary Table S2). For vector pCXUN-LbCpf1-OsU3-RCR1-RCR2-armed-DRT, among 94 plants recovered, PCR-RE and sequencing analyses identified two chimeric genotypes with one allele that had the expected precise gene replacement (Line 169-2 and 169-3), and five genotypes with partial replacements (Lines 168-2, 168-4, 168-5, 169-5, and 169-6) (Fig. 2B; Supplementary Table S2). Different genotypes observed in these lines indicated that they were independent lines. No off-target effects were observed at predicted potential off-target sites in these tested lines (Table S3).

## Discussion

### Cpf1-induced DSBs enable targeted gene replacement in rice

We precisely replaced the wild-type *ALS* gene with the intended mutant version that carries two discrete point mutations conferring herbicide resistance to rice plants through HDR of LbCpf1-induced DSBs by using DRT either with only a left homologous arm or with both homologous arms (Fig. 2). The achievement of precise gene replacement events using only left armed-DRT not only further our understanding of the potential mechanism underlying HDR of DSBs, but also greatly simplifies the design of DRTs for precision genome editing in crop improvement. However, we observed a relatively lower efficiency of CRISPR/Cpf1-mediated *ALS* gene replacement in this study, compared with that of CRISPR/Cas9-mediated gene replacement in our previous studies (Sun *et al.*, 2016; J. Li *et al.*, 2018). The difference in efficiency probably results from the fact that the editing activity of Cpf1 is not as high as that of Cas9 as reported by others and our previous study (Ma *et al.*, 2015; Wang *et al.*, 2017; S. Li *et al.*, 2018). Despite the lower efficiency, CRISPR/LbCpf1-mediated gene replacement targets sequences that cannot be edited by Cas9 due to the differences in PAM requirement (Jinek *et al.*, 2012; Cong *et al.*, 2013). LbCpf1 utilizes a thymidine-rich PAM ‘TTTV’ (Zetsche *et al.*, 2015), which enables targeting AT-rich regions of a genome such as 5'- and 3'-untranslated regions and the promoter of a gene of interest. It is usually more difficult to find suitable target sequences in the above-mentioned regions for SpCas9, which has a PAM requirement of NGG. Therefore, CRISPR/Cpf1-mediated gene editing is particularly useful for precisely knocking-in a gene of interest or replacing a gene/promotor at a targeted specific locus, or inserting a small DNA fragment that encodes an epitope tag or fluorescence protein at the 5' or 3' end of a gene.

### HDR of Cpf1-induced DSBs in rice is consistent with the proposed SDSA model

When a dsDNA DRT is used as the repair template, the Cas9-(D10A) induced 5' overhang DNA structure and DRTs with an asymmetric left homology arm are particularly amenable to gene conversion/replacement (Bothmer *et al.*, 2017). Cpf1 cleavage produces 5'-protruding sticky ends, which may facilitate the pairing and insertion of the DRT through microhomology-mediated end joining (MMEJ) (Zetsche *et al.*, 2015). In this study, we added two crRNA targets at each end of the DRTs. We achieved either precise gene replacement or partial HDR events both in transient assays and in stable edited rice plants (Figs 1B, C, 2). However, we did not detect any events harbouring the integration of the entire DRT with the target sequences at each end, indicating that the DSBs are indeed resected through exo- and/or endonucleolytic processing of the 5' end to yield the 3' overhangs on both sides of the DSBs (Bothmer *et al.*, 2017; Paix *et al.*, 2017). The invasion of 3' end single strands may lead to the formation of a Holliday junction (HJ) and paired with the homologous arms of the DRT, and then extended by DNA synthesis, thus resulting in conservative precise gene replacement (Puchta, 1998). Our observations of the successful achievement of HDR events when a DRT with only the left homologous arm was used as repair template are consistent with a replicative repair process that requires pairing between a 3'-homology arm on the donor and homologous sequences in the genome on at least one side of the DSBs, as demonstrated recently in human cells (Paix *et al.*, 2017). Besides, we also observed the template switching between DRT and wild-type sequences as shown in some partial HDR events (Figs 1B, C, 2), indicating the occurrence of template switching due to unstable replication forks during multiple rounds of dissociation and re-invasion (Smith *et al.*, 2007). This phenomenon, again, is similar to the recently reported precision gene editing using Cas9 endonuclease in human cells (Paix *et al.*, 2017).

Taken together, we here demonstrate synthesis-dependent repair of Cpf1-induced DSBs, which enables us to replace the wild-type *ALS* gene precisely with the intended mutant version that carries two discrete point mutations conferring herbicide resistance to rice plants. Our finding that donor with only the left homology arm is sufficient to achieve targeted gene replacement enables us to gain a better mechanistic understanding of HDR of DSBs for targeted gene replacement in various organisms and simplifies the design of DRTs to improve agriculturally important traits in crops through CRISPR/Cpf1-mediated genome editing.

## Supplementary data

Supplementary data are available at *JXB* online.

Fig. S1. The map of plasmids used in this study.

Fig. S2. Sequences of the DRTs.

Table S1. The primer sets used in this study.

Table S2. Characterization of regenerated rice plants in the T_0_ generation.

Table S3. Analysis of potential off-target effects.

## Author contributions

LQX and YDZ conceived the project. SYL, JYL, JHZ, WMD, JDF and SS performed the experiments. LQX and YDZ wrote the manuscript.

## Supplementary Material

Supplementary Figures and Tables
