# Supplementary material for: Synthesis-dependent repair of Cpf1-induced double strand DNA breaks enables targeted gene replacement in rice
Source: J Exp Bot. 2018 Jun 28;69(20):4715–21. doi: 10.1093/jxb/ery245 (PMC6137971; doi:10.1093/jxb/ery245)
Supplement: Supplementary Figures and Tables [file ery245_suppl_supplementary_figures_tables.pdf]

Supplementary Data

Synthesis-dependent repair of Cpf1-induced double-strand DNA breaks enables targeted gene replacement in rice

Shaoya Li[1], Jingying Li[1], Jiahui Zhang[1], Wenming Du[1], Jindong Fu[1], Suhas Sutar[1], Yunde Zhao[2, 3, *] and Lanqin Xia[1, *]

Contents

Fig. S1 The map of plasmids used in this study

(A) pCXUN-LbCpf1-OsU3-RCR1-RCR2-left-armed-DRT

(B) pCXUN-LbCpf1-OsU3-RCR1-RCR2-armed-DRT

Fig. S2 Sequences of the DRTs

(A) DRT with only left homology arm

(B) DRT with two homology arms

Table S1 The primer sets used in this study

Table S2 Characterization of regenerated rice plants in T_0 generation

Table S3 Analysis of potential off-target effects

A

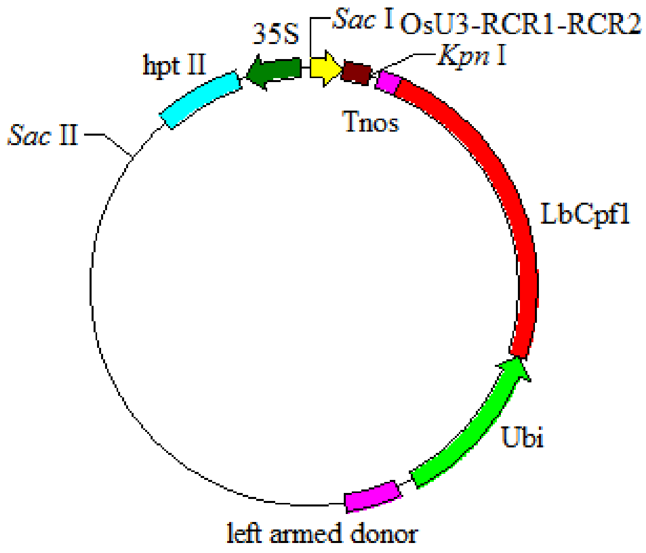

pCXUN-LbCpf1-OsU3-RGR1-RGR2-left-armed-DRT

B

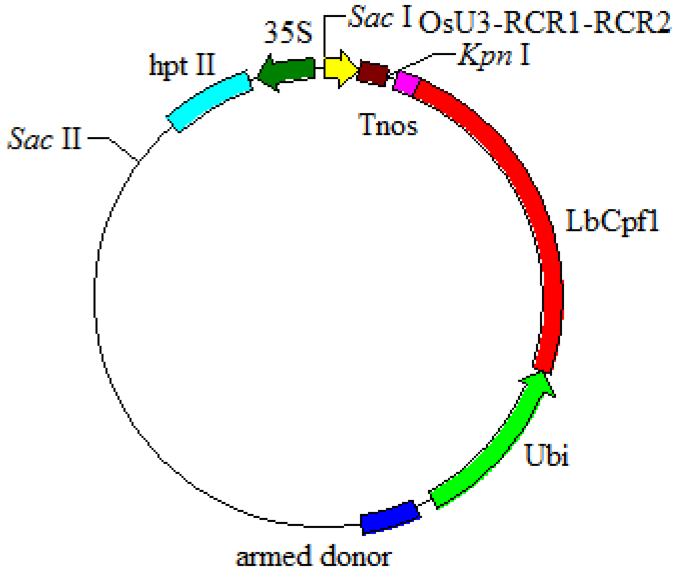

pCXUN-LbCpf1-OsU3-RGR1-RGR2-armed-DRT

Fig. S1. A schematic show of the vectors used in this study

(A) pCXUN-LbCpf1-OsU3-RGR1-RGR2-left-armed-DRT

(B) The map of pCXUN-LbCpf1-OsU3-RGR1-RGR2-armed-DRT

A

Target 1 (23 bp) Left arm (97 bp)

AACCAACATTTGGGTATGGTGGTGCAATGGGAGGATTGATGGGGATGGTAGCTTCCTCATGAACATTCAGGAGCTGGCATTGATCCGCATTGAGAACCTCCCTGTGAAGGTGATGG

TGTTGAACAACCAACAccTaGGcATGGTcGTcCAgTtGGAGGATAGGTTTTACAAGGCGAATAGGGCGCATACATACTTGGGCAACCCGGAATGTGAGAGCGAGATATATCCAGATTTT

Mutated target 1 Mutated G to T: W-L Mutated *Eco*RV

GTGACTATTGCTAAGGGGTTCAATATTCCTGCAGTCCGTGTAACAAAGAAGAGTGAAGTCCGTGCCGCCATCAAGAAGATGCTCGAGACTCCAGGGCCATACTTGTTGGAcATCATC

GTCCCGCACCAGGAGCATGTGCTGCCTATGATCCCAAtTGGGGGCGCATTCAAGGACATGATCCTGGATGGTGATGGCAGGACTGTGTATTAATCTATAATCTGTATGTTGGCAAAGC

Mutated G to T: S-I

ACCAGCCCGGCCTATGTcTGACgTGAATGACtCATAAAGAGTGTTTGACCTGAATGACCCATAAAGAGTGGTATGCCTA

Mutated target 2 Target 2 (23 bp)

B

Target 1 (23 bp) Left arm (97 bp)

AACCAACATTTGGGTATGGTGGTGCAATGGGAGGATTGATGGGGATGGTAGCTTCCTCATGAACATTCAGGAGCTGGCATTGATCCGCATTGAGAACCTCCCTGTGAAGGTGATGG

TGTTGAACAACCAACAccTaGGcATGGTcGTcCAgTtGGAGGATAGGTTTTACAAGGCGAATAGGGCGCATACATACTTGGGCAACCCGGAATGTGAGAGCGAGATATATCCAGATTTT

Mutated target 1 Mutated G to T: W-L Mutated *Eco*RV

GTGACTATTGCTAAGGGGTTCAATATTCCTGCAGTCCGTGTAACAAAGAAGAGTGAAGTCCGTGCCGCCATCAAGAAGATGCTCGAGACTCCAGGGCCATACTTGTTGGAcATCATC

GTCCCGCACCAGGAGCATGTGCTGCCTATGATCCCAAtTGGGGGCGCATTCAAGGACATGATCCTGGATGGTGATGGCAGGACTGTGTATTAATCTATAATCTGTATGTTGGCAAAGC

Mutated G to T: S-I

ACCAGCCCGGCCTATGTcTGACgTGAATGACtCATAAAGAGTGGTATGCCTATGATGTTTGTATGTGCTCTATCAATAACTAAGGTGTCAACTATGAACCATATGCTCTTCTGTTTTACTT

Mutated target 2 Right arm (121 bp)

GTTTGATGTGCTTGGCATGGTAATCCTAATTAGCTTCCTGCTGTTTGACCTGAATGACCCATAAAGAGTGGTATGCCTA

Target 2 (23 bp)

Fig. S2. Sequences of the DRT with only left homology arm and the DRT with two homology arms

(**A**) DRT with only left homology arm; (**B**) DRT with two homology arms. The sequences shadowed in yellow are derived from the wild-type *ALS* gene sequence, sequences of two targets are underlined, the PAM and mutated PAM sequences are boxed, the left and right arms are shadowed in blue, the mutated *Eco*RV target site are shadowed in gray, the mutated amino acids are shadowed in green, and the mutated synonymous changes in the donor are in red lower case.

Table S1. The primer sets used in this study

Primer	Sequence（5′ to 3′）	Use of PCR products	Annealing (℃)	Fragment size (bp)
RGR1F2<br>RGR-common-R	TAATTTCTACTAAGTGTAGATGGTATGGTGGTGCAATGGGAGGAGGCCGGCATGGTCCCAGCCT<br>GTCCCATTCGCCATGCCGAAGC	Construct vector for RGR1	58	112
RGRF1<br>RGR-common-R	AAATTACTGATGAGTCCGTGAGGACGAAACGAGTAAGCTCGTCTAATTTCTACTAAGTGTAGAT<br>GTCCCATTCGCCATGCCGAAGC	Construct vector for RGR1and RGR2	58	155
RGR2-F2<br>RGR-common-R	TAATTTCTACTAAGTGTAGATACCTGAATGACCCATAAAGAGTGGGCCGGCATGGTCCCAGCCT<br>GTCCCATTCGCCATGCCGAAGC	Construct vector for RGR2	58	112
OsU3F<br>OsU3R<br>OsU3-RGR1R	AAGGAATCTTTAAACATACGAACAGATCA<br>TGCCACGGATCATCTGCAC<br>CACGGACTCATCAGTAATTTTGCCACGGATCATCTGCAC	Construct vector for OsU3-RGR1-RGR2	56	404
RGR-Common-F<br>RGR1-10 random-R	AAATTACTGATGAGTCCGTGAGGAC<br>GTAATTTGGTCGTATTCGTCCCATTCGCCATGCCG	Construct vector for OsU3-RGR1-RGR2	58	172
RGR2-10 random-F<br>Sac-RGR2R	CGAATGGGACGAATACGACCAAATTACTGATGAGTCCGTGAGGAC<br>CCCTTTCGCCAGGGGTACCGGTCCCATTCGCCATGCCGAAGC	Construct vector for OsU3-RGR1-RGR2	58	195
Sac-OsU3F<br>Sac-RGR2R	GATTACGAATTCGAGCTCAAGGAATCTTTAAACATACGAACAGATCA<br>CCCTTTCGCCAGGGGTACCGGTCCCATTCGCCATGCCGAAGC	Construct vector for OsU3-RGR1-RGR2	58	739
Pme-donorF<br>Pme-donorR	GTTTCCCGCCTTCAGTTTAAACGTAAAACGACGGCCAGT<br>GTCAAACACTGATAGTTTAAACCAGGAAACAGCTATGAC	Construct vector for donor	56	873

donor-armLF<br>donor-armLR1	AACCAACATTTGGGTATGGTG<br>CATAGGCCGGGCTGGT	Construct vector for donor (with only left arm)	60	486
donor-armLF<br>donor-armLR2	AACCAACATTTGGGTATGGTG<br>CACTCTTTATGGGTCATTCAGGTCAAACATAGGCCGGGCTGGT	Construct vector for donor (with only left arm)	58	522
pme-donor-armLF<br>pme-donor-armLR	GTTTCCCGCCTTCAGTTTAAACAACCAACATTTGGGTATGGTG<br>GTCAAACACTGATAGTTTAAACCACTCTTTATGGGTCATTCAGGT	Construct vector for donor (with only left arm)	58	566
ALSTestF<br>T2MR	GGCAATCATCGCTACTGGTG<br>CACTCTTTATGAGTCATTCACGTCAG	Detect edited *ALS* gene in calli	58	648
ALSTestF<br>ALSTestR	GGCAATCATCGCTACTGGTG<br>ATGCCTACAGAAAACAACACACTACA	Detect edited *ALS* gene in plants	58	803
hptF<br>hptR	GAGGGCGTGGATATGTCCTG<br>ATTGACCGATTCCTTGCGGT	Detect *hptII* gene in plants	58	306
U3F<br>U3R	AAGGAATCTTTAAACATACGAACAGATC<br>TTTGGATCCTCTAGAGATTATGTGG	Detect crRNA sequence in plants	57	509
LbCpf1F<br>LbCpf1R	GCCTGTTGCACAAGGATTAGACTTC<br>GGAGCAGAGCTGTTCATGAGG	Detect LbCpf1 transgene in plants	58	684
ALS-OFF1-F<br>ALS-OFF1-R	GCTGCTCTGTTGTCTGGGTC<br>ACGAGCAACAGCCAGAATCA	Detect mutations on putative off-target site of *ALS* target 1	59	800

ALS-OFF2-F<br>ALS-OFF2-R	GGTTGCAAAGTCACATGCGTG<br>TCCGGAGATTCCTGACGTGAG	Detect mutations on putative off-target site of *ALS* target 1	59	818
ALS-OFF3-F<br>ALS-OFF3-R	GGTTTACCTTTCTAGCGCACTTC<br>CGATGTTCTTAGGCGCACC	Detect mutations on putative off-target site of *ALS* target 1	59	658
ALS-OFF4-F<br>ALS-OFF4-R	CACCCATAAGGAATGGGTCTTC<br>GCTCTTGATTGGGCCGGC	Detect mutations on putative off-target site of *ALS* target 2	57	999
ALS-OFF5-F<br>ALS-OFF5-R	CTACCTAGGGCAGCTGCTG<br>CAGAGCCATCCCAGACTTTAC	Detect mutations on putative off-target site of *ALS* target 2	59	524

Table S2.Characterization and genotyping of regenerated plants in T_0 generation

Vector	NO. of calli for transform	NO. of resistant calli	NO. of individual rice plants recovered	Line	Genotype
pCXUN-LbCpf1-OsU3-RGR1-RGR2-left-armed-DRT	152	15	71	171-2	Heterozygous HR
				171-9	chimeric HR
				170-1<br>172-1	Partial HR
pCXUN-LbCpf1-OsU3-RGR1-RGR2-armed-DRT	164	20	94	169-2	chimeric HR
				169-3	
				168-2	Partial HR
				168-4	
				168-5	
				169-5	
				169-6	
				169-1	Random indels

Table S3. Analysis of potential off-target effects

Target	Name of putative off-target site	Putative off-target locus	Sequence of the putative off-target site	No. of m ismatching b ases	No. of plants sequenced	No. of plants with mutations
Target 1	ALS1-OFF1	chr02:4712511-4712537	TTTAGAAATTGTTGTGCAAGGGGAGGA	5	12	12
	ALS1-OFF2	chr05:26016305-26016331	TTTGGGTAAGATCATGCAAAGGGAGAA	6	12	12
	ALS1-OFF3	chr03:33738572-33738598	TTTGATTTTGGTGGTGGAATGGAAGAA	6	12	12
Target 2	ALS2-OFF1	chr08:4284947-4284973	TTTCACCTAAATAACCCATTAGGAGTA	5	12	12
	ALS2-OFF2	chr07:584165-584191	TTTGCCCTGGATGGCCCTTTAAGAGGG	6	12	12

Note: The PAM motif is underlined; mismatching bases are shown in red.
